# Nanostructured Boron Nitride With High Water Dispersibility For Boron Neutron Capture Therapy

**DOI:** 10.1038/srep35535

**Published:** 2016-10-19

**Authors:** Bikramjeet Singh, Gurpreet Kaur, Paviter Singh, Kulwinder Singh, Baban Kumar, Ankush Vij, Manjeet Kumar, Rajni Bala, Ramovatar Meena, Ajay Singh, Anup Thakur, Akshay Kumar

**Affiliations:** 1 Advanced Functional Materials Lab., Department of Nanotechnology, Sri Guru Granth Sahib World University, Fatehgarh Sahib, 140 407, Punjab, India; 2 Central Scientific Instruments Organization, Chandigarh, 160 030, India; 3 Department of Physics, Amity School of Applied Science, AMITY University Haryana, Gurgaon, 122 413, India; 4 Defence Institute of Advanced Technology (DU), Pune, 411 025, India; 5 Department of Mathematics, Punjabi University, Patiala, 147 002, Punjab, India; 6 Nanotoxicology laboratory, School of Environmental Sciences, Jawaharlal Nehru University, New Delhi, 110 067, India; 7 Technical Physics Division, BARC, Mumbai, 400 085, India; 8 Department of Basic and Applied Sciences, Punjabi University, Patiala, 147 002, Punjab, India

## Abstract

Highly water dispersible boron based compounds are innovative and advanced materials which can be used in Boron Neutron Capture Therapy for cancer treatment (BNCT). Present study deals with the synthesis of highly water dispersible nanostructured Boron Nitride (BN). Unique and relatively low temperature synthesis route is the soul of present study. The morphological examinations (Scanning/transmission electron microscopy) of synthesized nanostructures showed that they are in transient phase from two dimensional hexagonal sheets to nanotubes. It is also supported by dual energy band gap of these materials calculated from UV- visible spectrum of the material. The theoretically calculated band gap also supports the same (calculated by virtual nano lab Software). X-ray diffraction (XRD) analysis shows that the synthesized material has deformed structure which is further supported by Raman spectroscopy. The structural aspect of high water disperse ability of BN is also studied. The ultra-high disperse ability which is a result of structural deformation make these nanostructures very useful in BNCT. Cytotoxicity studies on various cell lines (Hela(cervical cancer), human embryonic kidney (HEK-293) and human breast adenocarcinoma (MCF-7)) show that the synthesized nanostructures can be used for BNCT.

Boron nitride (BN) exists in cubic, rhombohedral and hexagonal forms similar to carbon materials. Cubic is analogous to diamond with similar hardness. Rhombohedral exists rarely as in case of carbon^1^ and hexagonal boron Nitride is equivalent to graphite^2^. Hexagonal boron nitride is one of the old powder metallurgical product shows outstanding electrical and thermal properties^3^. This material also wraps itself to form nanotubes. These nanotubes have improved properties as compared to carbon nanotubes in respect of their band gap. Band gap of BN nanotubes is independent of tube diameter^4^. Hexagonal BN exhibit a good resistance to corrosion, low density, higher melting point and excellent chemical stability^5^ which renders this material as a prominent candidate for Boron Neutron Capture Therapy (BNCT)^6^ in cancer treatment. Various *in vitro* and *in vivo* studies confirmed that Boron Nitride materials have shown better biocompatibility and lower cytotoxicity than their carbon counterparts^7^,^8^,^9^,^10^,^11^. One of the main challenges in respect to integration of nanostructures of BN into various biological systems was their poor suspension/hydroxylation in various biological solutions^12^,^13^,^14^. Various methods were tried to improve suspension ability/hydroxylation of these materials like surface functionalization^15^,^16^ and wrapping by other molecules or interactions^17^,^18^,^19^,^20^,^21^,^22^,^23^. But these methods were unable to give desired results. One of the other major reasons for lacking of research on this material was synthesis conditions which include relatively very high temperature (1400 °C). Research on nanotubes of boron nitride and carbon started on the same year^24^, but these tough synthesis conditions leave the research behind as compared to carbon^25^. Two different atom in the BN structure with electro negativity difference of about one unit make this material partial polar in nature more useful as compare to carbon based materials^26^,^27^. This structural variation can be exploited for many remarkable applications. Till date many synthesis routes were tried to synthesize nanostructured boron nitride like, arc discharge^28^,^29^,^30^ ball milling^31^,^32^,^33^,^34^,^35^,^36^,^37^, chemical vapour deposition(CVD)^38^,^39^,^40^,^41^,^42^. The main Limitations of these routes are as follows; i. Arc discharge and CVD are relatively high temperature synthesis routes, ii. Contamination is the main limitation of ball milling. Recently, pulsed laser plasma deposition technique^43^ is also used for synthesis of boron nitride nanotubes/nanosheets. The limitation of the process is its specific instrumentation. The last major drawback of all the synthesis routes was that; none of them was able to deliver a product with ultra-high water disperse-ability. This limits the use of the synthesized materials for biological applications. Ultra-high water disperse-ability of the as synthesized boron nitride can be used in biological applications^44^. Present study also deals with structural (crystallographic) property (solubility) relation of the material. A relatively low temperature single step synthesis route is used for synthesis of nanostructured BN. The as synthesized nanostructures need no further purification and are ready to use for different applications. Cytotoxicity studies were also tested on various cell lines (Hela cells (cervical cancer), Human embryonic kidney (HEK-293) and human breast adenocarcinoma (MCF-7)) which showed that the material is suitable for BNCT.

## Experimental

Boric Acid (*H*_3_*BO*_3_) and Ammonia were used as initial ingredients as source of boron and nitrogen respectively. The average particle size and purity of boric acid powder were 20 *μ*m and 99.9%, respectively. For present investigations, a specially designed stainless steel (304) autoclave is used. In typical experiments, boric acid and ammonia were put in an autoclave of 50 ml capacity in the ratio 15 (g):40 (ml) respectively. The charged autoclave was heated at 700 °C and 10 (GPa)^45^ pressure for 24 h. After cooling, the white solid powders were taken out from the autoclave. The powders were dried at 50 °C for 5 h in a vacuum heating oven. The proposed reaction which may have occurred in the autoclave is written as


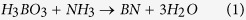


### Cytotoxicity assay

Cytotoxicity assay studies, the cells were maintained in Iscove’s modified Dulbecco′s medium supplemented with 2 mM GlutaMAX, 100 *μ*g/ml streptomycin, 100 *μ*/ml penicillin and 10% Fetal calf serum (FCS) and incubated at 37 °C in an atmosphere of 95% air and 5% *CO*_2_ at 90% relative humidity. Cytotoxic effect on the Hela (cervical cancer), Human embryonic kidney (HEK-293) and human breast adenocarcinoma (MCF-7) cell lines was also assessed. Briefly, 5X10^3^ cells/well were incubated in 100 *μ*l of RPMI-1640 supplemented with 10% FCS, 2 mM l-glutamine and various concentrations of Boron nitride nanostructures. The cytotoxic effects of boron nitride nanostructures were tested using a standard MTT (3-(4,5-Dimethylthiazol-2-yl)-2,5-diphenyltetrazolium bromide) assay, in a 96-well microtiter plate for 24 hrs and 48 hrs. MTT is a non-radioactive assay done routinely to assess the viability of the cell culture. After the incubation period, 20 *μ*l of MTT dye solution (5 mg/ml in Phosphate-buffered saline(PBS) pH 7.4) was added to each well. After 4 hrs of further incubation the formazan crystals formed by the cellular reduction of MTT were dissolved in 150 *μ*l of Dimethyl Sulfoxide (DMSO) and plates were read on an ELISA-reader using 570 nm filter. All measurements were done in triplicates. The relative cell viability (%) related to control wells containing cells without nanostrutures was calculated by


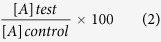


where [A] test is absorbance of the test sample and [A]control is the absorbance of the control sample. By using the non-radioactive assay for assessing the proliferation of cells, we were able to quantify the amount of MTT cleaved, which is directly proportional to the viable cell population.

### Characterization

All the samples were characterized by X-ray diffraction (XRD) using Pan Analytical X-ray diffractometer with CuK*α* radiation (*λ* = 1.5418 Å). Microstructural examinations of samples were performed using a high-resolution transmission electron microscope(HRTEM) (TEM, Model TECNAI 20G2 F TWIN) and field emission scanning electron microscope (FESEM) (FESEM, Sigma Carl Zeiss). Micro-Raman analysis was done by using RENISHAW spectrometer. Zeta potential determination was done using Dynamic Light Scattering (Zetasizer Nano ZS, Malvern Instruments Ltd., UK) method. UV-visible spectrum was obtained using UV-Visible spectrophotometer (Shimadzu UV-2600). Fourier transform infrared spectroscopy (FTIR) analysis was performed using FTIR spectrometer (Bruker Alpha). Differential thermal analysis (DTA) and thermo gravimetric analysis (TGA) were carried out using Hitachi STA-7300.

#### Observation of Cellular Morphology 

To observe the changes in cellular morphology, Hela cells were plated into a 12-well plate overnight. Following treatment described in previous section for 24 hrs, the cells were washed with PBS and visualized under an Olympus CKX41 phase contrast microscope (Olympus, Tokyo, Japan) to detect morphological changes such as cellular shape, chromatin condensation, intracellular vesicles and multinucleated cells.

## Results and Discussion

**Figure 1:**
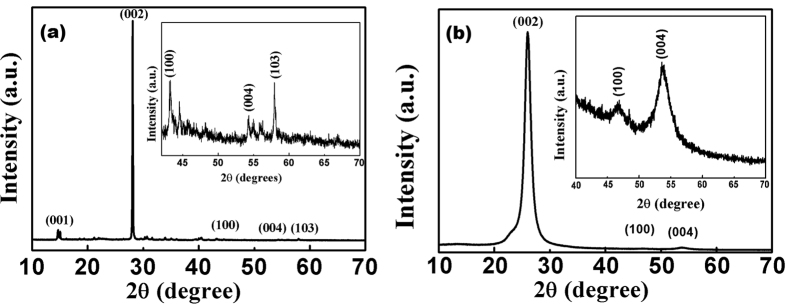
(**a**) XRD pattern of boron nitride powder (Sample 1). (**b**) XRD pattern of boron nitride solution in water (Sample 2).

The decomposition of ammonia in the used synthesis route generates hydrogen which is responsible for boron nitride formation even at relatively low temperature. The generated hydrogen played an important role in the nucleation of BN^46^. XRD patterns of the synthesized powder (Fig. 1(a)) shows that the synthesized product is boron nitride (ICDD 01-07-32095). Critical analysis of XRD showed that the synthesized powder is highly crystalline in nature and highly textured along (002) plane with texture coefficient 1.99. Texture coefficient^47^ is calculated using the relation given below


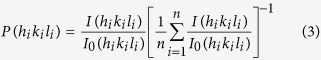


where P (hkl) is texture coefficient of the plane specified by miller indices, *I* (hkl) and *I*_0_ (hkl) are the specimen and standard intensities respectively for a given peak and n is the number of different peaks.

**Figure 2:**
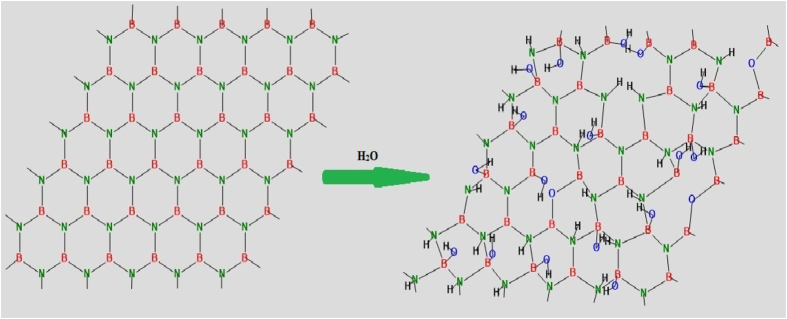
Hydroxylation of boron nitride sheets.

**Figure 3:**
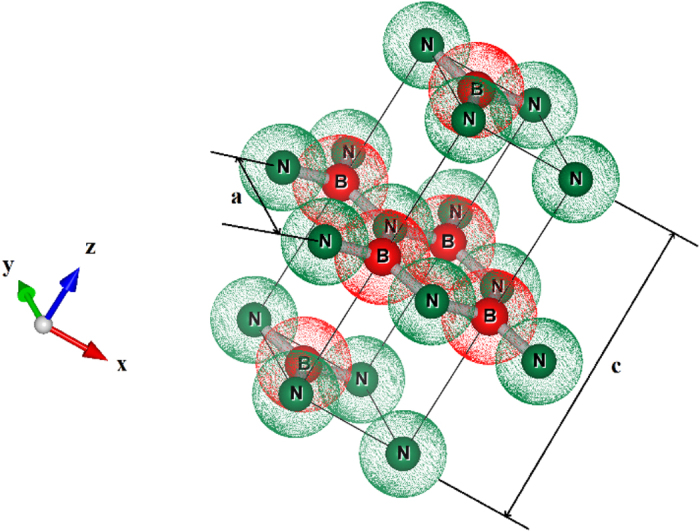
Deformed hexagonal boron Nitride crystal structure with calculated lattice parameters; a = 2.0896 Å, c = 6.0205 Å generated by Vesta software^50^.

**Figure 4:**
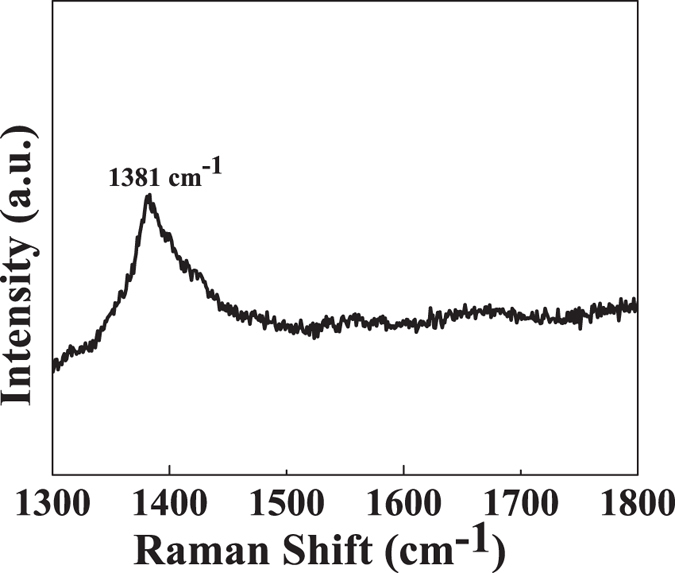
Raman Spectra of boron nitride.

Also from texture analysis, we can conclude that material is grown along least dense plane. The structure is relatively less compact as compare to normal structure so its chemical properties may vary from its compact counterpart^48^. In an ideal texture of this kind, the grain orientation of sheets are fixed with respect to axis of the sheet^49^. The lattice parameter analysis (done by XRD) shows that the structure is under compressive strain with about 16% decrease in ‘a’ value and about 10% decrease in ‘c’ value (shown in Table 1). This non uniform change in ‘a’ and ‘c’ values distort the crystal structure and ultimately the electron clouds of the atoms, making the structure relatively unstable and in compressive strain. The as synthesized powder was suspended in distilled water. The suspension was again analysed by XRD using special accessories (Fig. 1(b)). It was observed that BN retains its crystallinity even in water (ICDD 00-04-50896). It seems that the material dissociate to form a clear solution. Dissociation leads the structure to relatively relaxed state (Fig. 2). The structure (Fig. 3) is elongated along ‘c’ to overcome the compressive strain in the XRD pattern which may be the possible reason of its dispersion and forming a clear solution. The other peaks are relatively small so to clearly distinct these peaks an inset magnified view is used. Zeta potential analysis was also done on the sample which shows that the material is stable with zeta potential −16.2 mV (Figures S1 and S2)^51^. †Raman band was observed at 1381 *cm*^−1^ which is attributed to Raman active mode *E*_2*g*_ due to in plane atomic displacement of boron and nitrogen atom against each other (Fig. 4)^52^. The shift in the peak to higher wave number is due to compressive strain present in the system which is also verified by XRD analysis (decreases in ‘a’ and ‘c’ values). Raman peaks are associated with the lattice vibration and hence are affected by atomic bond and chemical structure of the material. Thus, stress present in the structure affected the Raman peak^53^,^54^. The crystalline structure and ordering of hexagonal BN layered were evaluated by Raman shift and full width at half maximum (fwhm) of the *E*_2*g*_ vibrational band. It is well known fact that, high quality single crystal (h-BN) shows an intrinsic *E*_2*g*_ vibration peak at 1367 *cm*^−1^ with 9.1 *cm*^−1^ as fwhm^55^. *E*_2*g*_ shifts to higher frequencies for polycrystalline materials. Domain size is also related to fwhm of this peak, fwhm increases with decrease in size^56^,^57^. In present case the blue shifted *E*_2*g*_ vibration mode (1381 *cm*^−1^) is due to weaker interaction between inter-layer of BN. The broadening of *E*_2*g*_ peak may be attributed to size shrinking of ordered BN layers^58^.

**Figure 5:**
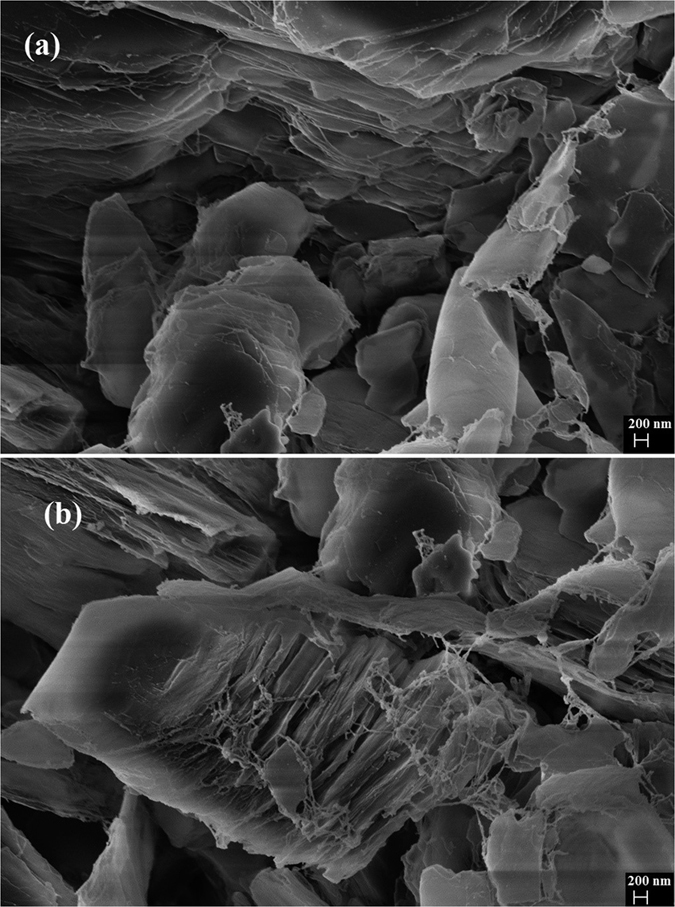
(**a**) Flakes of nanostructured Boron Nitride and (**b**) Growth of Boron nitride nanotubes from flakes.

FESEM images (Fig. 5(a,b)) revels that the structure is layered with B-N layers stacked over each other. This layered structure have very high surface area with electronegative atoms in the structure, also the structure is under compressive strain so the these flakes of B-N starts rolling along the axis to form BN nanotubes (Fig. 5(b)).

**Figure 6:**
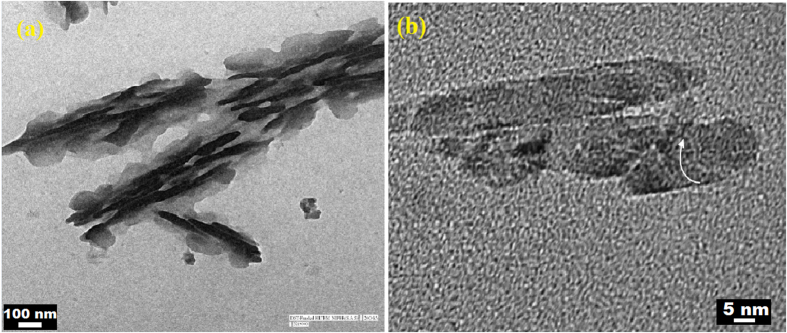
(**a**) Flakes of nanostructured Boron Nitride and (**b**) Wrapped Boron Nitride flakes to form Boron Nitride nanotubes.

TEM analysis also support the above FESEM analysis, Fig. 6(a) shows that the two dimensional layered structure of Boron Nitride. These layers are combing and further wrapping to form BN nanotubes (Fig. 6(b)). Microstructural analysis showed that the material is having layers similar to graphene sheets.

**Figure 7:**
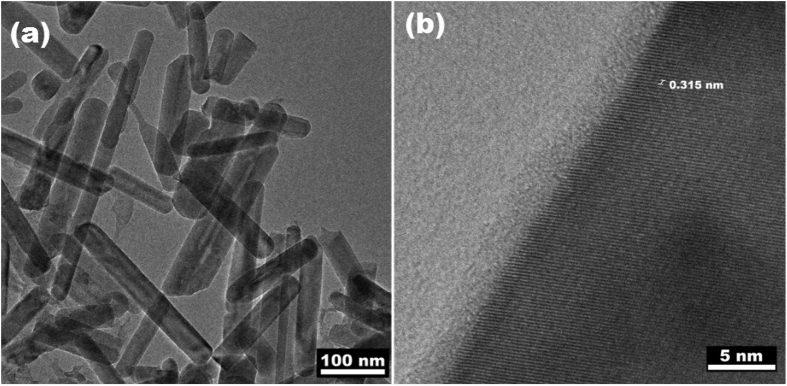
(**a**) Boron Nitride nanotubes and (**b**) Highly grown (002) plane lattice fringes.

Figure 7(a) also confirms the formation of nanotubes†. Lattice fringes confirm the growth of (002) plane with 0.315 nm as ‘d’ value (Fig. 7(b)). Similar structural features and planes are also reported by other researches^59^,^60^. The theoretical analysis of the structure has also been done using Virtual Nano Lab software. The structure is generated for as synthesized sample having lattice parameters(a = 2.0896 Å, c = 6.0205 Å) and space group (P-6m2). †The simulated structure was then wrapped to form nanotubes and further changes were observed as in TEM and SEM images. The theoretical band gap (VNL Software) results are well matched with the experimentally calculated band gap using UV-visible spectroscopy (Figures S3 and S4) †.

**Figure 8:**
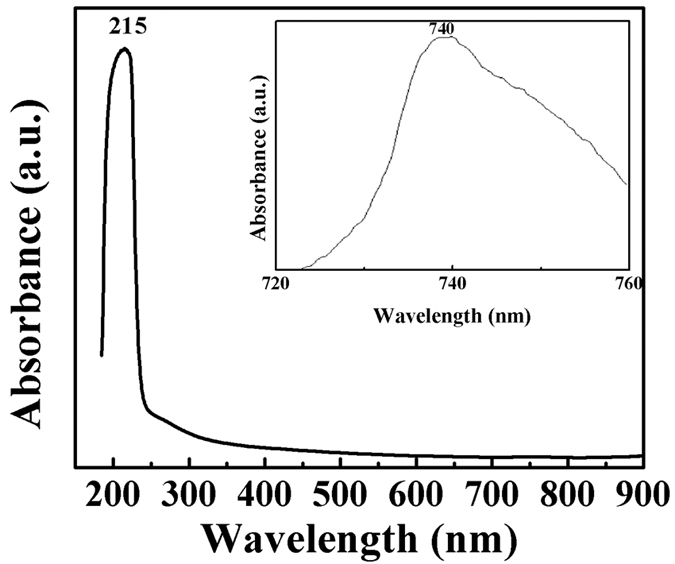
UV visible spectra of boron nitride.

The UV-visible spectrum displays one sharp absorption peak at 215 nm (Fig. 8). The optical band gap of boron nitride is approximately 5.7 eV, calculated using Tauc plot. Figures S3 and S4, concluded that the band gap is in good approximation with the theoretically calculated band gap value (5.5 eV)†. When the sample was examined in 700–800 nm wavelength range a hump at about 740 nm was also observed. This hump is attributed to BN nanotubes. The calculated band gap for this peak is about 1.7 eV. The theoretically calculated band gap for nanotubes of similar diameter is about 2.05 eV. The crystal structure is less stable having high energy state so it can be hydroxylated easily, which is the possible reason of its high dispersibility in water.

**Figure 9:**
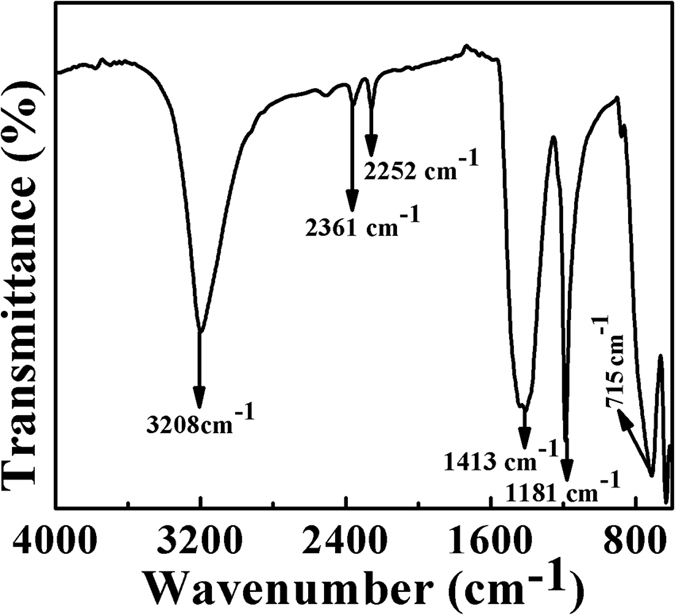
FTIR Spectrum of boron nitride.

FTIR spectrum (Fig. 9) peaks were observed at 715, 1181, 1413 and 3208 *cm*^−1^ due to B-N, BN-O bonding, attribution to hexagonal BN and B-OH bonding, respectively^61^. The FTIR spectra confirms the formation of hexagonal BN.

**Figure 10:**
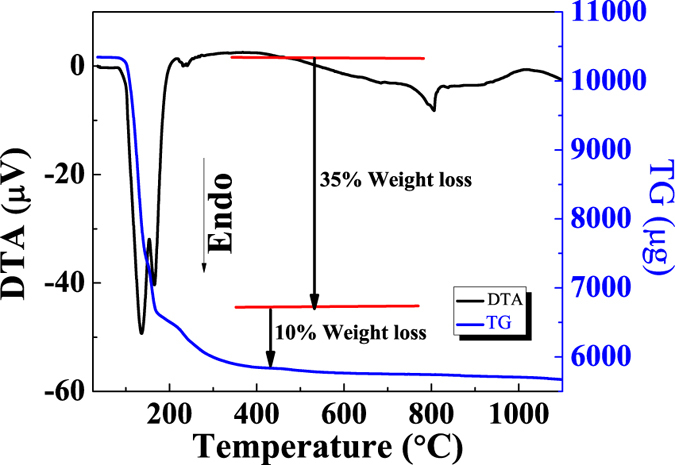
TG and DTA curve of boron nitride.

The differential thermal analysis (DTA) and thermogravimetry (TG) curves are illustrated in Fig. 10. TG analysis showed a continuous weight loss up to 200 °C, the corresponding changes are also reflected in DTA curve. This may be attributed to evaporation of water molecules. After 300 °C the line shows the thermal stability of boron nitride even at high temperature^62^.

### Cytotoxicity analysis

**Figure 11:**
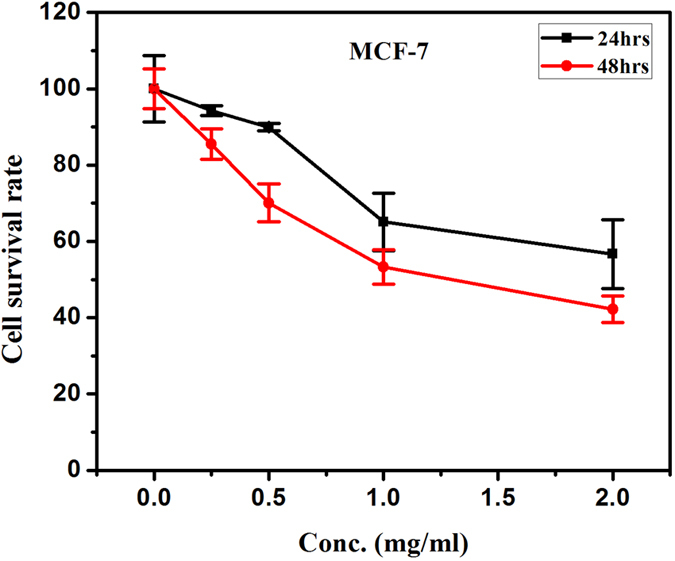
Cytotoxic effects of boron nitride on MCF-7 cell lines.

**Figure 12:**
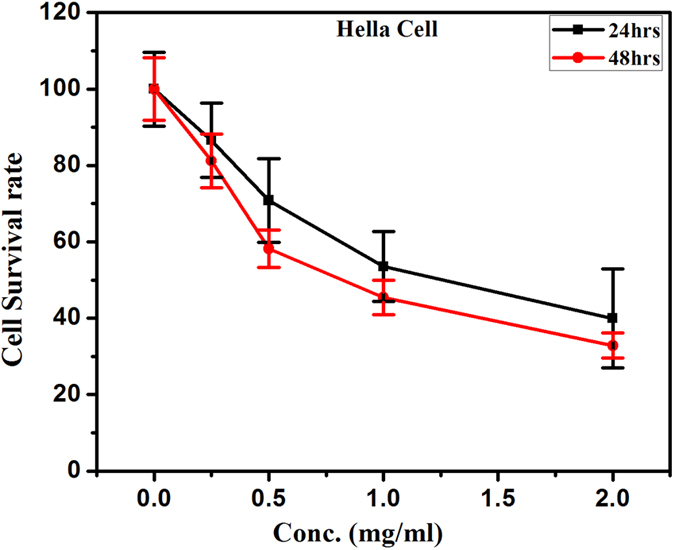
Cytotoxic effects of boron nitride on Hela cell lines.

**Figure 13:**
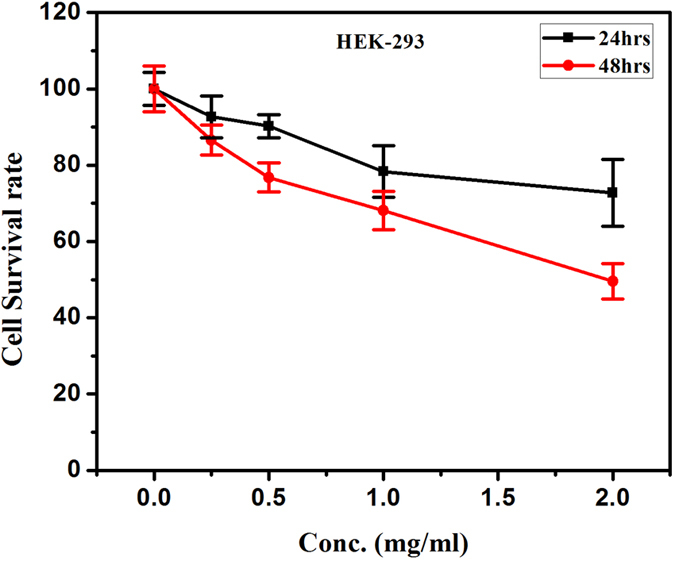
Cytotoxic effects of boron nitride on HEK-293 cell lines.

The MTT assay is widely used for its fast, inexpensive, and simple procedure for screening viability in large number of samples; however, the assay is subject to variability and does not discriminate between the routes of cell death. The cell viability of cancerous and normal cells decreased as a function of dose and time. Cancerous cell lines, MCF-7 and Hela showed 45 and 60% cytotoxicity respectively at a dose of 2 mg/ml for 24 hrs treatment, which further increased up to 60 and 70% respectively in 48 hrs treatment (Figs 11 and 12). Whereas in normal cell line (HEK-293) 30% cytotoxicity was observed at higher dose 2 mg/ml and 24 hrs treatment, which increases up to 50% in later period (48 hrs) shown in Fig. 13. It is evident that the cytotoxicity of BN nanostructures is more in cancerous cells as compared with normal cell lines.

This is an interesting observation that haemopoeitic cells are more susceptible to BN nanostructures. We also tested the efficacy of the BN nanostructures in p53 Hela, and p53 mutated breast cancer cell line MCF-7 which clearly gave us different results. In normal cells (HEK-293) around 30% cytotoxicity was observed at a higher dose (~2 mg/ml) of BN nanostructures treatment. But, the intact cell morphology can be correlated with no loss of membrane integrity and reduced cytotoxicity. The more membrane pore size may be responsible for enhanced uptake of BN nanostructures leading to cytotoxicity. The above observations warrant further investigation into the mode of cell death.

## Conclusion

**Figure 14:**
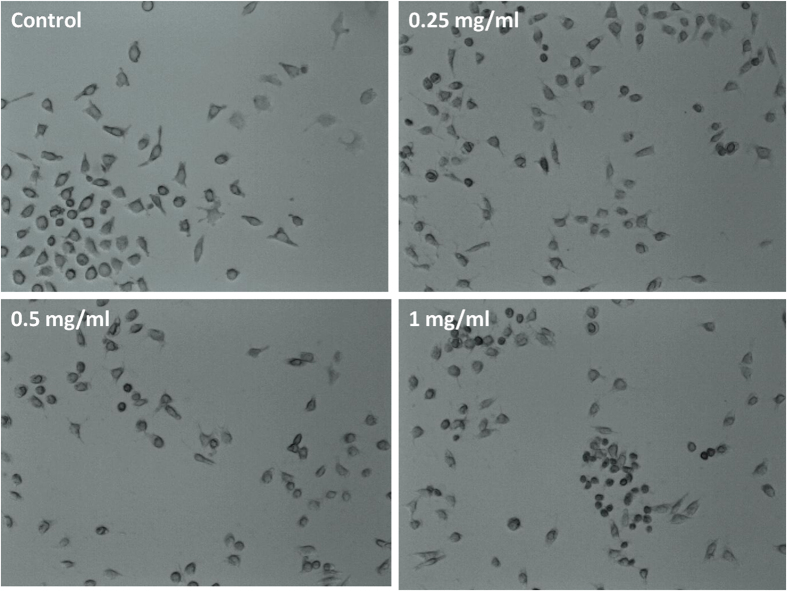
Cellular morphology of Hela (cervical cancer) cell lines with different doses of boron nitride.

Highly water dispersible nanostructured BN can be synthesized at relatively low temperature by modifying the synthesis techniques. The hexagonal BN is rather open structure as compare to its cubic counterpart type so it can be compressed. The synthesized material which showed dual band gap and thermal stableness can be useful in optical as well as high temperature applications. Also high dispersibility in water makes this material useful in BNCT. To confirm whether or not these nanostructures can be further applied in living cell imaging, the Cytotoxicity of BN nanostructures has been examined. At lower doses (0.25 mg/ml), we did not observed any remarkable changes in cellular morphology, however with increasing dose the cell come under stress as seen in Fig. 14 (1 mg/ml). These results shows that lower doses of BN nanostructures can be used in biomedical applications.

## Additional Information

**How to cite this article**: Singh, B. *et al*. Nanostructured Boron Nitride With High Water Dispersibility For Boron Neutron Capture Therapy. *Sci. Rep.* **6**, 35535; doi: 10.1038/srep35535 (2016).

## Supplementary Material

Supplementary Information

## Figures and Tables

**Table 1:** XRD Analysis.

Sample label	Calculated (Å)	Actual (Å)	Lattice Parameter Change (%)	c/a ratio (calculated)	c/a ratio (Actual)	Plane (hkl)	Texture Coefficient
Sample1	a = 2.0896	a = 2.5040	16.5	2.88	2.66	002	1.9999
	b = 2.0896	b = 2.5040	16.5			100	0.1148
	c = 6.0205	c = 6.6612	9.60			103	1.8865
Sample2	a = 2.2600	a = 2.5000	9.60	3.00	2.60	002	0.8018
	b = 2.2600	b = 2.5000	9.60			100	0.0315
	c = 6.8000	c = 6.6609	2.00			004	0.1670
