# Supplementary material for: Nanostructured Boron Nitride With High Water Dispersibility For Boron Neutron Capture Therapy
Source: Sci Rep. 2016 Oct 19;6:35535. doi: 10.1038/srep35535 (PMC5069687; doi:10.1038/srep35535)
Supplement: Supplementary Information [file srep35535-s1.pdf]

Nanostructured Boron Nitride With High Water Dispersibility For Boron Neutron Capture Therapy

Bikramjeet Singh[1], Gurpreet Kaur[1], Paviter Singh[1],Kulwinder Singh[1], Baban Kumar[2], Ankush Vij[3], Manjeet Kumar[4], Rajni Bala[5], Ramovatar Meena[6], Ajay Singh[7], Anup Thakur[8], Akshay Kumar *[1]

[1]Advanced Functional Materials Lab., Department of Nanotechnology,
Sri Guru Granth Sahib World University, Fatehgarh Sahib-140 407, Punjab, India
[2]Central Scientific Instruments Organization, Chandigarh-160 030, India
[3]Department of Physics, Amity School of Applied Science, AMITY University Haryana, Gurgaon-122 413, India
[4]Defence Institute of Advanced Technology (DU), Pune-411 025, India
[5] Department of Mathematics, Punjabi University, Patiala-147 002, Punjab, India
[6]Nanotoxicology laboratory, School of Environmental Sciences, Jawaharlal Nehru University, New Delhi-110 067, India
[7]Technical Physics Division, BARC, Mumbai- 400 085, India
[8]Department of Basic and Applied Sciences, Punjabi University, Patiala-147 002, Punjab, India

*Corresponding author: akshaykumar@gmail.com

Dispersibility of Boron Nitride nanostructures in water at different concentration:

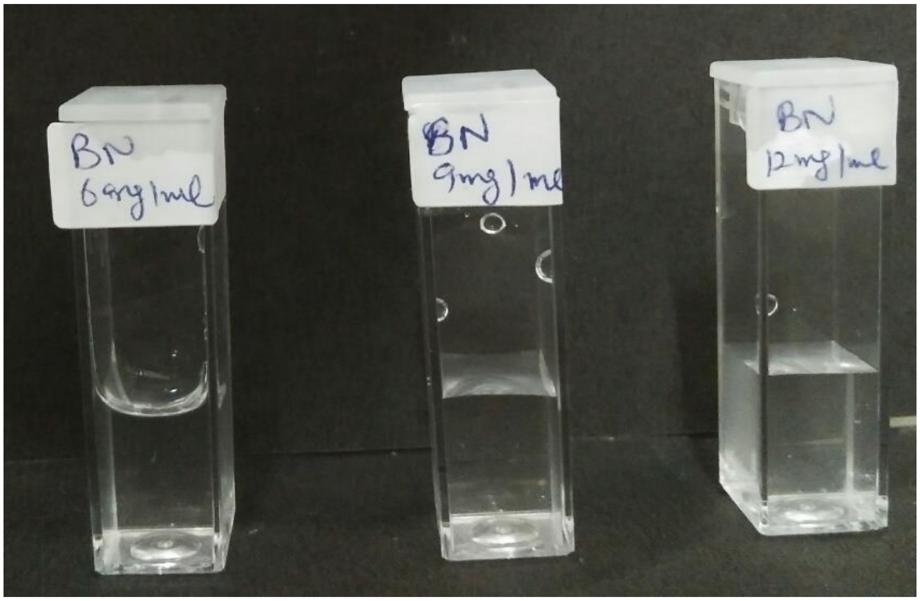

Figure S1: Dispersibility of Boron Nitride nanostructures in water at different concentration

Zeta potential determination of Boron Nitride nanostructures:

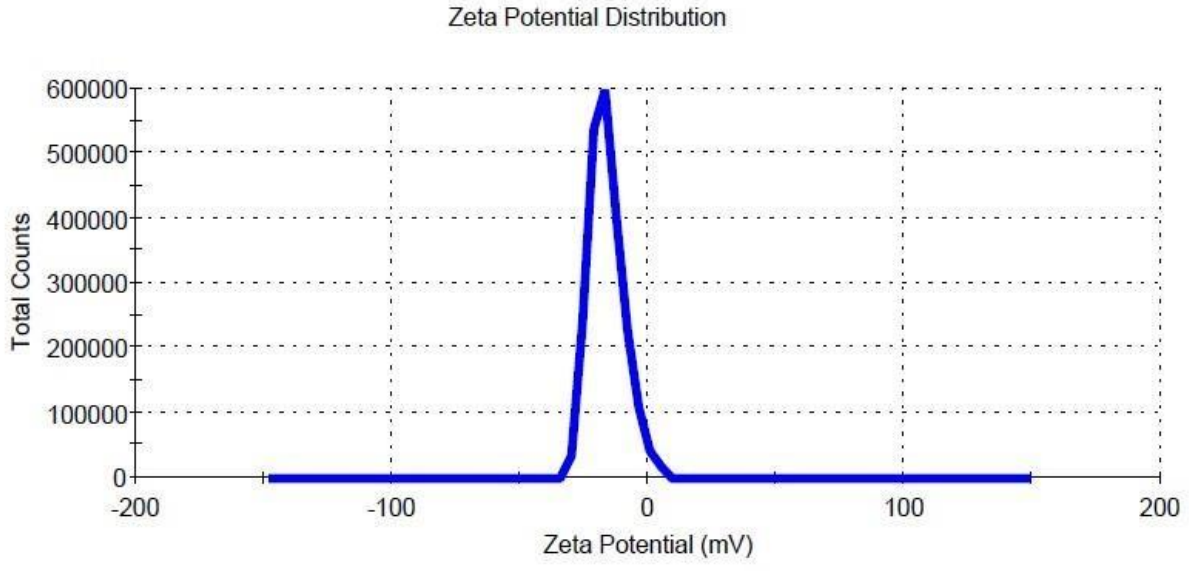

Figure S2: Zeta potential of Boron Nitride

Theoretical study on band gap determination of boron nitride nanosheet and nanotube:

Theoretical studies of as synthesized material were done by Virtual Nano Lab (VNL) software. These structures were generated by VNL software. The calculated lattice parameters from X-ray diffraction (XRD) analysis were used. These tubes have same diameters as approximated by transmission electron microscope (TEM). The calculated band gap is in well approximation with experimental results.

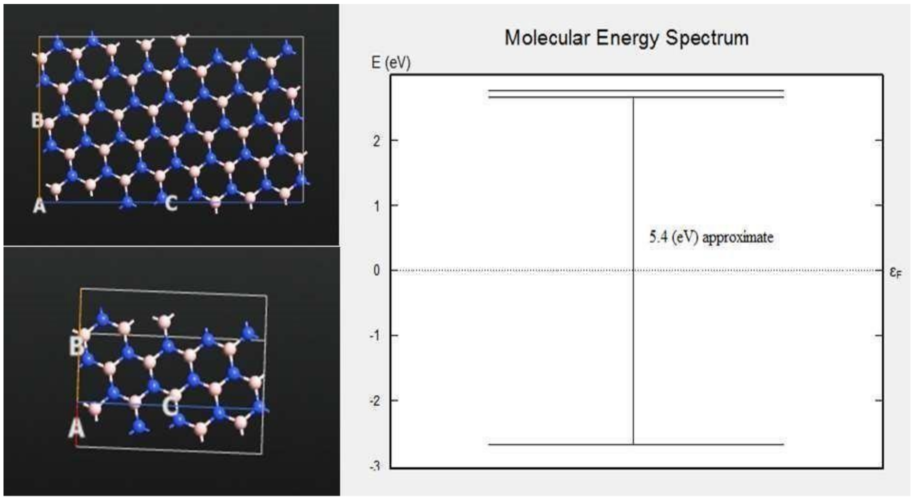

Figure S3: Boron Nitride Nanosheet and its band gap

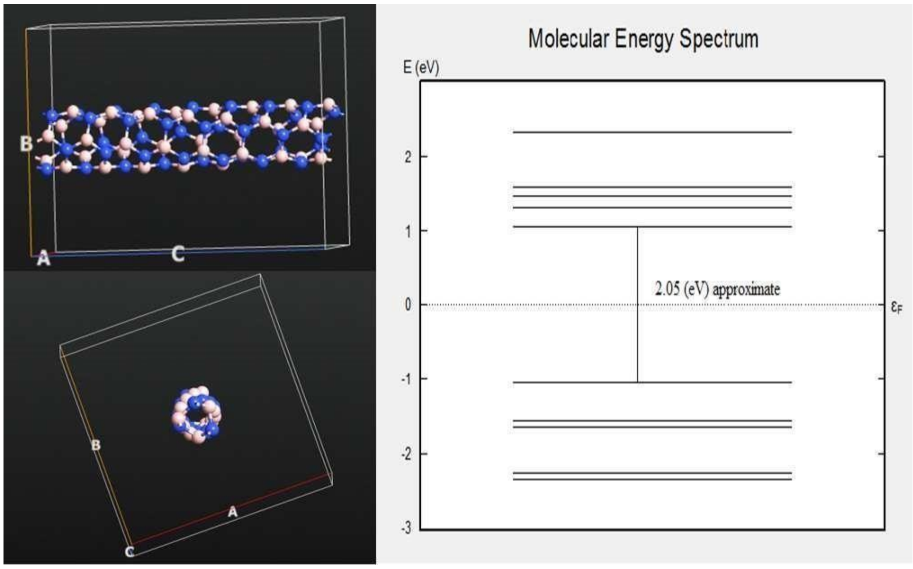

Figure S4: Boron Nitride Nanotube and its band gap

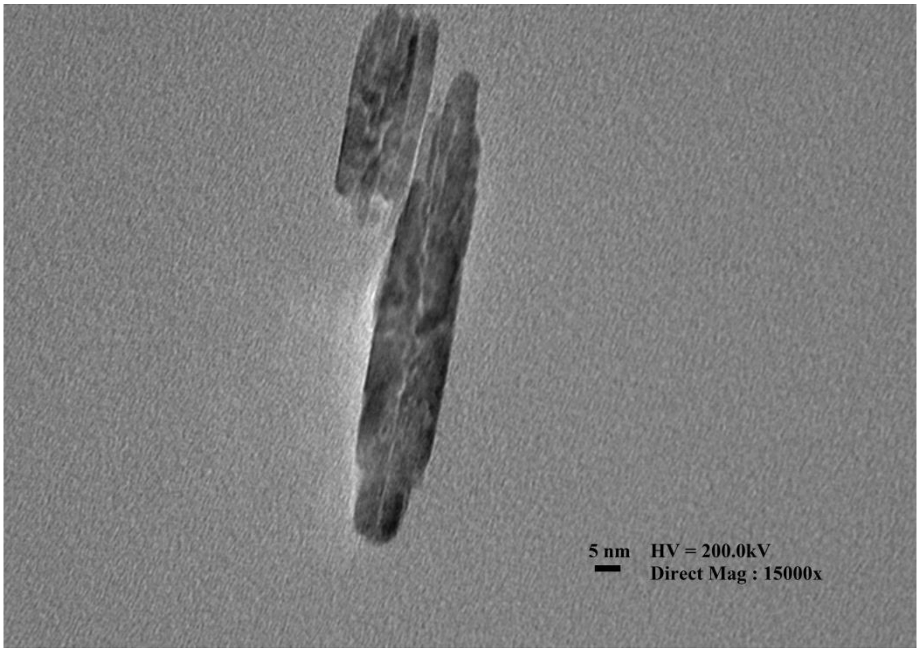

Figure S5: Wrapping of flaks to form boron nitride nanotubes
